# Mortality differences between ICUs that are regarded as ‘in control’: a longitudinal register-based study in the Netherlands, 2013–2023

**DOI:** 10.1136/bmjopen-2025-107572

**Published:** 2026-04-20

**Authors:** Fabian Termorshuizen, Sylvia Brinkman, Sesmu M Arbous, Dave A Dongelmans, Nicolette F de Keizer, Ferishta Bakhshi-Raiez

**Affiliations:** 1 National Intensive Care Evaluation (NICE) Foundation, Amsterdam, The Netherlands; 2 Department of Medical Informatics, Amsterdam UMC Locatie AMC, Amsterdam, The Netherlands; 3 Quality of Care, Amsterdam Public Health Research Institute, Amsterdam, The Netherlands; 4 Department of Intensive Care Medicine, Leiden University Medical Center, Leiden, The Netherlands; 5 Department of Clinical Epidemiology, Leiden University Medical Center, Leiden, The Netherlands; 6 Department of Intensive Care Medicine, Amsterdam UMC Locatie AMC, Amsterdam, The Netherlands

**Keywords:** INTENSIVE & CRITICAL CARE, Mortality, Quality in health care

## Abstract

**Abstract:**

**Objectives:**

Funnel plots are used to identify intensive care units (ICUs) with a higher than expected risk-adjusted mortality. ICUs with a standardised mortality ratio (SMR) within pre-defined control limits (often the 99.8% CL) are regarded as ‘in control’ and not labelled as a potential outlier for a particular calendar year. However, increased mortality rates not due to random fluctuations within and across the calendar years may be overlooked. We examined whether statistically significant and relevant differences in mortality over time between ICUs regarded as ‘in control’ are present.

**Design:**

A longitudinal register-based study.

**Setting and participants:**

88 ICUs in the Netherlands registering the admissions of all critically ill patients in the National Intensive Care Evaluation registry in the Netherlands from 2013 to 2023.

**Primary outcome measure:**

Hospital death analysed in a multivariable logistic regression analysis with a random intercept for ICU. The random intercept variance was translated to the median OR (MOR).

**Results:**

877 ICU-calendar year combinations were included, covering 759 498 unique admissions. The MOR increased from 1.12 (95% CI 1.10 to 1.15) for ICU-calendar year combinations with an SMR within the narrowest 95% CL (N=677) to 1.20 (1.17 to 1.24) for combinations with an SMR within the expanded 99.8% CL (including adjustment for overdispersion) (N=194) and to 1.21 (1.17 to 1.25) when including all ICU-calendar year combinations. Similar results were found for separate calendar years and separate diagnostic groups.

**Conclusions:**

These results show differences in mortality between ICUs that were not labelled as outliers. Assessment of mortality performance should integrate cross-sectional funnel plots, the MOR and longitudinal trends in the SMR to better capture persistent patterns of excess risk.

**STRENGTHS AND LIMITATIONS OF THIS STUDY**

- A large and high-quality database was used with more than 750 000 intensive care unit (ICU) admissions from more than 80 ICUs covering 11 calendar years.
- The calculations of the standardised mortality ratios (SMRs) and control limits of the funnel plots were done according to the procedures used in the daily registry practice of the National Intensive Care Evaluation and other quality registries.
- Demonstration of variation in performance beyond annual outlier detection by the estimation of the median OR and the graphical display of the SMRs of an individual ICU during a number of subsequent calendar years is easy to implement in the daily registry practice.
- A high SMR, even over several successive years, does not necessarily imply impaired quality of care, but requires further careful and detailed inspection.
- Factors at the ICU level that may explain persistent variation were not investigated.

## Introduction

 Reporting outcomes at an institutional level is important for quantifying, comparing and finally improving the quality of healthcare.^1^ Patient-level data are routinely collected in various and increasing numbers of clinical quality registries. Hospital mortality is the most common outcome for evaluating intensive care unit (ICU) performance.^2^ The standardised mortality ratio (SMR), defined as the ratio of observed to the expected number of hospital death events of a particular ICU with its specific case-mix in a particular calendar year, is often used as the metric of primary interest for benchmark studies.^3^ The expected numbers of death events are derived from a risk-adjustment model such as the Acute Physiological And Chronic Health Evaluation-IV, which accounts for illness severity and case-mix for a given ICU in a specific calendar year. The position of the SMR relative to the control limits (CL) in the so-called funnel plot is decisive for qualifying a unit as ‘in-control’ versus ‘out of control’, that is, the observed number is according to expectation versus not. If not, the ICU may be regarded as a potential high or low outlier, and a detailed examination is needed before drawing a conclusion in terms of bad versus good quality of care for that ICU. These CL are typically drawn by using the 95%-probability limits, and may be expanded by using 99.8%-probability limits and by adjusting for overdispersion.^4^

The potential reputational damage and employment consequences for healthcare providers who are publicly labelled as high outliers are significant. Consequently, clinical registries often hesitate to cast a wide net to detect as many potential underperformers as possible at the cost of lower specificity.^5^ By using more widely spaced CL, the risk of misclassifying a healthcare provider, such as an ICU, with a true ‘underlying’ SMR of 1.00 and an outlier position that is purely due to random fluctuation or to correctable data issues becomes very small. However, such practice may lead to a lower sensitivity and potentially overlook ICUs with mortality rates that require further scrutiny.^6^

In the present study, we use data from the Dutch National Intensive Care Evaluation (NICE) registry. Since 2013, NICE registry has published funnel plots for the SMR for separate calendar years, covering the total ICU population as well as separate diagnostic subgroups.^7^ This is done to monitor the quality of care.^8^ This study aimed to find out whether statistically significant and clinically relevant differences in hospital mortality can be observed among those ICUs that are regarded as ‘in control’, that is, ICUs not labelled as potential high or low outliers. This was done by analysing the position of the SMRs of a specific ICU for subsequent calendar years.

## Methods

### Data

In 1996, NICE registry started the collection of data on ICU admissions in the Netherlands. When an ICU participates, all its admissions are recorded. Since 2016, all Dutch ICUs have participated. Demographic, clinical and laboratory data are collected to make the calculation of the Acute Physiological And Chronic Health Evaluation (APACHE)-IV mortality risk and case-mix adjustment possible.^9^ ICU and hospital length of stay and ICU and hospital death are included in the registry. The purpose is to provide feedback on performance indicators to ICUs in order to monitor their quality of care. To ensure that the data are of high quality, the data are subjected to automated quality checks, onsite data quality audits take place and data collectors participate in training sessions.^10^ In case of missing data on individual items of the APACHE-IV model, this was taken into account using the guideline for the calculation of the APACHE-IV. Often, missingness was due to a lack of clinical indication to assess a lab test, and zero points should be assigned for that item.

All admissions recorded in NICE database with admission dates in the years 2013–2023 were extracted. The data of ICUs related to periods of insufficient data quality, as determined by onsite data quality audits, were excluded. Furthermore, those admissions not under the final responsibility of an intensive care physician (such as recovery or coronary care unit patients) and admissions not fulfilling the APACHE-IV inclusion criteria were excluded (see online supplemental figure 1).^9^

### Patient and public involvement

Patients or the public were not involved in the design, conduct, reporting or dissemination plans of this research.

### Statistical analysis

#### Standardised mortality ratio

For an ICU in a particular calendar year, the SMR was calculated by dividing the observed number by the expected number of hospital deaths. The calendar year was chosen as the unit of reporting time, as the aim of the study was to follow the usual registry practice of NICE (and many other quality registries).

The expected number of hospital deaths was calculated by adding up the estimated APACHE-IV mortality risks associated with the individual ICU admissions for that ICU in the pertinent calendar year. The APACHE-IV model was recalibrated in a logistic regression model on hospital death as outcome and the logit-transformed non-recalibrated APACHE-IV risk, calendar year and terms for the interaction of {calendar year×APACHE-IV risk} as predictors. This was done separately for coronary artery bypass grafting (CABG) and non-CABG patients as APACHE-IV handles different models for these two groups. Analyses were conducted for all included admissions, and separately for the so-called ‘Cardiac surgery group’ and ‘Non-cardiac surgery group’ (see online supplemental table 1). Analyses were also performed separately for several specific diagnostic subgroups (see online supplemental table 1).

#### Control limits

Two pairs of CL were calculated. The first pair was based on the 95% probability limits of a Poisson distribution with the expected number of deaths (x-axis) as the parameter for the definition of both the expectation and the variance.^6^ As the Poisson distribution is related to discrete numbers, the exact positions of the upper and lower 95% CL were determined by applying an interpolation method. For the 95% CL, the interpolation resulting in a narrower funnel was used by allowing p values higher than the nominal probability of 5% (for details, see Manktelow and Seaton^11^). The second pair of CL was based on the 99.8% probability limits.^6^ For these limits, the interpolation method resulting in a wider funnel was used by allowing p values less than the nominal probability of 0.2% (for details, see Manktelow and Seaton^11^). Possible overdispersion, that is, when the variance appears to be higher than expected given a Poisson distribution, was taken into account by calculating a so-called Winsorised estimate of the variance inflation factor.^4 12^ This was done for each separate calendar year and, if higher than 1.00, used to further expand the 99.8% CL for that calendar year by multiplying the variance with this factor.

In the analysis of the total ICU population, the overdispersion factor appeared to be higher than 1.00 in all calendar years and the 99.8% CL were adjusted (widened) accordingly. However, for specific diagnostic subgroups, this factor was often lower than 1.00, indicating underdispersion (ie, the variance was lower than expected under the assumption of a Poisson distribution). If so, the 95% CL, not the 99.8% CL, was adjusted (narrowed) accordingly for that specific diagnostic subgroup in the pertinent calendar year. This was done to consistently follow the principle of contrasting the results when using the ‘narrowest 95% CL’ versus using the ‘widest 99.8% CL’.

It is Important to note that the CL were calculated according to the way this is done in the daily practice of the NICE registry, and especially the application and interpretation of the factors for overdispersion are a matter of debate (see the Discussion section). The Poisson distribution is used as the expected number of death events of a specific ICU is the benchmark for that ICU and this number fully defines the appropriate Poisson distribution, whereas specification of a binomial model requires two defining parameters (and, thus, two horizontal axes in the funnel plot): the probability and the number.

#### Categorisation of the ICU-calendar year combinations

The SMR of each ICU in a particular calendar year, positioned at the expected number of deaths on the x-axis, was compared with the applicable 95% CL and 99.8% CL for that calendar year. If the SMR was within the narrowest 95% CL, that ICU was considered as definitely ‘in control’ for that calendar year and, thus, certainly not labelled as a potential outlier. If the SMR was outside the 95% CL but inside the widest 99.8% CL, the ICU was also considered as ‘in control’, but only after adopting the more flexible criteria for being in control (see Control Limits), as is often done in practice. If the SMR was outside the widest 99.8% CL, that ICU was considered as definitely ‘out of control’ and labelled as a potential outlier. ICUs with an SMR outside the lower 95% CL or 99.8% CL may also be regarded as potential outliers, but in the favourable direction.

The analyses described in the next paragraph were performed in three variants: restricted to the selection of ICU-calendar year combinations with an SMR within the narrowest 95% CL (‘1. Narrow CL’), after expanding the selection by also including those with an SMR within the widest 99.8% CL (‘2. Expanded CL’), and by using the data of all combinations, including those that are outside the 99.8% CL (‘3. All’).

#### Median OR

Hospital mortality as outcome was analysed in a logistic regression model on individual-level patient data with adjustment for the APACHE-IV mortality risk (categorised in quintiles according to the selection included) and calendar year, and ICU included as a random intercept. The MOR is the median value of the distribution of ORs that are obtained by comparing the predicted mortality risks (ie, the mortality predicted by the logistic model, thus, not restricted to the APACHE-IV risk) within many pairs of randomly selected ICU patients with identical values for the fixed covariates in the model but admitted at two different arbitrarily selected ICUs.^13 14^ In each within-pair comparison, the patient with the highest predicted mortality risk is always placed in the numerator, the other in the denominator. This results in a range of possible ORs from 1.00 to infinity. As the difference between the predicted mortality risks of the two randomly selected patients within a pair can only be assigned to between-ICU differences, the MOR may be regarded as a valid indicator of between-ICU variation. The MOR was estimated by using an analytical formula in which the estimated variance of the random intercept is included (for derivation of this formula, see Yarnell *et al*^15^). The CI of the MOR was established by estimating the CI of the variance for the random intercept in a post-estimation command based on the profile likelihood method.^16^ This method implies a refitting of the model multiple times with different values for the estimated parameters, resulting in a range of values that are more or less likely as judged by the likelihood ratio test. A 95% CI for the MOR that does not include 1.00 shows statistical significance. Such a result implies that there are systematic differences in mortality between ICUs and, thus, room for improvement, even if the SMRs of these ICUs are definitely ‘in control’ and certainly not labelled as potential outlier (variant ‘1. Narrow CL’). Furthermore, the likelihood ratio test comparing a model with and a model without the variance for the random intercept was performed.

#### Graphical display of SMRs at the upper and lower edges of the distribution

The SMRs of an individual ICU were shown by calendar year in a graph, along with the 95% and 99.8% CL relevant for that ICU. This was done for those ICUs with a predicted random intercept at the highest 5% or lowest 5% quantile of the distribution of random intercepts, as estimated in the logistic regression analysis for variant ‘1. Narrow CL’. Thus, this analysis was restricted to ICUs with at least one SMR within the narrowest 95% CL. By visual inspection, it was examined whether there are ICUs with consistently increased or decreased SMRs for a number of subsequent years, but still regarded as definitely ‘in control’ for (a part of) these calendar years.

The statistical analyses were performed using the R statistical environment (V.4.3.2) (R Foundation for Statistical Computing, Vienna, Austria).

## Results

The selection (see online supplemental figure 1) resulted in an analysis file of 759 498 admissions at 88 ICUs, and 877 ICU-calendar year combinations.

In table 1, the numbers of ICU-calendar year combinations, unique admissions and observed hospital deaths are given for the different SMR categories delineated by the 95% and 99.8% CL, which were defined for each calendar year in 2013–2023 separately. This is done for SMRs related to all admissions, and separately for SMRs related to admissions in the ‘Cardiac surgery group’ and to all other admissions (in the ‘Non-cardiac surgery group’) (see also online supplemental table 1).

**Table 1:** The number of ICU-calendar year combinations, the associated numbers of admissions and hospital deaths

	Total	Categories for the SMR delineated by the CL				
		<99.8% CL low	<95% CL low	>95% CL low to <95% CL high	>95% CL high	>99.8% CL high
		N (%)	N (%)	N (%)	N (%)	N (%)
**2013–2023**						
All diagnoses						
ICU-calendar year combinations						
N	877	8	100	671	94	4
Row %		0.9	11.4	76.5	10.7	0.5
Admissions						
N	759 498	15 595	106 531	524 675	107 247	5450
Row %		2.1	14	69.1	14.1	0.7
Hospital death events						
N	92 828	1090	9681	66 285	14 960	812
Column %		7	9.1	12.6	13.9	14.9
Non-cardiac surgery group						
ICU-calendar year combinations						
N	877	6	101	672	94	4
Row %		0.7	11.5	76.6	10.7	0.5
Admissions						
N	612 105	5097	72 505	438 207	92 514	3782
Row %		0.8	11.8	71.6	15.1	0.6
Hospital death events						
N	89 572	625	8994	63 854	15 309	790
Column %		12.3	12.4	14.6	16.5	20.9
Cardiac surgery group						
ICU-calendar year combinations with expected number of deaths >1						
N	168	2	24	108	33	1
Row %		1.2	14.3	64.3	19.6	0.6
Admissions						
N	146 779	1700	23 466	93 623	27 329	661
Row %		1.2	16	63.8	18.6	0.5
Hospital death events						
N	3223	24	447	1957	772	23
Column %		1.4	1.9	2.1	2.8	3.5

Each SMR associated with an ICU-calendar year combination was categorised according to the position of the SMR relative to the pertinent 95% and 99.8% control limits (CL). The CL were defined for each calendar year in 2013–2023 separately. This was done for all admission diagnoses, and separately for the ‘Cardiac surgery group’ and ‘Non-cardiac surgery group’ (see the Methods section).

ICU, intensive care unit; SMR, standardised mortality ratio.

The SMRs (related to all admission diagnoses) of the majority of ICU-calendar year combinations were within the narrowest 95% CL (N=671, 76.5%). The SMRs of a small minority were outside the widest 99.8% CL, N=8 (0.9%) at the lower (<99.8% CL low) and N=4 (0.5%) at the upper part (>99.8% CL high) of the distribution. The SMRs of the remaining part (N=194, 22.1%) were outside the 95% CL and within the 99.8% CL. As expected, the observed hospital mortality increased from the lowest SMR category <99.8% CL low (7.0%) to the highest SMR category >99.8% CL high (14.9%).

The distribution over the categories defined by the 95% and 99.8% CL was reasonably consistent over the calendar years (see online supplemental table 2), with the exception of the most extreme parts (<99.8% CL low and >99.8% CL high) that were often lacking because of zero observations. In online supplemental figure 2, the funnel plot for 2019 including all admissions with the two pairs of CL is given.

### Median OR

In table 2, the results of the logistic regression on patient-level data are shown. In variant ‘1. Narrow CL’ (all diagnoses), the MOR was 1.126 (1.104 to 1.155), showing significant differences in hospital mortality between ICUs that were definitely not labelled as outliers. When expanding the selection in variant ‘2. Expanded CL’, that is, also including those ICU-calendar combinations with an SMR between the 95% CL and 99.8% CL, the MOR increased to 1.205 (1.172 to 1.248). When including all data in variant ‘3. All’, that is, including also the extreme outliers outside the 99.8% CL, the MOR was 1.212 (1.179 to 1.256). The analysis related to admissions in the ‘Non-cardiac surgery group’ yielded similar results (table 2). The analysis related to admissions in the ‘Cardiac surgery group’ showed slightly higher MORs (eg, for variant ‘1. Narrow CL’: 1.146 (1.075 to 1.261)). Similar results were found for separate calendar years (online supplemental table 3).

**Table 2:** The median OR (MOR) as estimated from a logistic regression model on hospital death with adjustment for the Acute Physiological And Chronic Health Evaluation-IV mortality risk and calendar year, and with intensive care unit (ICU) included as random intercept

	Variant ‘1. Narrow CL’		Variant ‘2. Expanded CL’		Variant ‘3. All’	
	MOR	(95% CI)	MOR	(95% CI)	MOR	(95% CI)
2013–2023						
All diagnoses	1.126	(1.104 to 1.155)	1.205	(1.172 to 1.248)	1.212	(1.179 to 1.256)
Likelihood ratio test	χ^2^=424.2, df=1, p<0.001		χ^2^=1388.4, df=1, p<0.001		χ^2^=1619.3, df=1, p<0.001	
Non-cardiac surgery group	1.118	(1.096 to 1.146)	1.205	(1.172 to 1.248)	1.213	(1.179 to 1.257)
Likelihood ratio test	χ^2^=299.9, df=1, p<0.001		χ^2^=1277.5, df=1, p<0.001		χ^2^=1494.9, df=1, p<0.001	
Cardiac surgery group	1.146	(1.075 to 1.261)	1.267	(1.18 to 1.434)	1.294	(1.2 to 1.478)
Likelihood ratio test	χ^2^=13.3, df=1, p<0.001		χ^2^=125.2, df=1, p<0.001		χ^2^=167.3, df=1, p<0.001	

The analysis was performed at first with restriction to the data of those ICU-calendar year combinations with a standardised mortality ratio (SMR) within the 95% CL (control limits) (variant ‘1. Narrow CL’), next with expansion of the selection with ICU-calendar year combinations with an SMR outside the 95% CL but inside the 99.8% CL (variant ‘2. Expanded CL’) and finally using all data (variant ‘3. All’). For all admission diagnoses, and separately for the ‘Cardiac surgery group’ and ‘Non-cardiac surgery group’ (see the Methods section).

Differences in the MOR across specific diagnostic groups were found (online supplemental table 4). High MORs were found in the analysis of admissions due to out-of-hospital cardiac arrest, malignancy and elective surgery (for variant ‘1. Narrow CL’: 1.201, 1.312 and 1.205, respectively). In some other diagnostic subgroups, such as trauma (1.074 (1.00 to 1.157)) and stroke (1.036 (1.00 to 1.114)), low and non-significant MORs were found in the analysis for variant ‘1. Narrow CL’, but the MORs increased to substantial and significant values after extending the selection in variants ‘2. Expanded CL’ and ‘3. All’.

### Graphical display of SMRs at the upper and lower edges of the distribution

**Figure 1:**
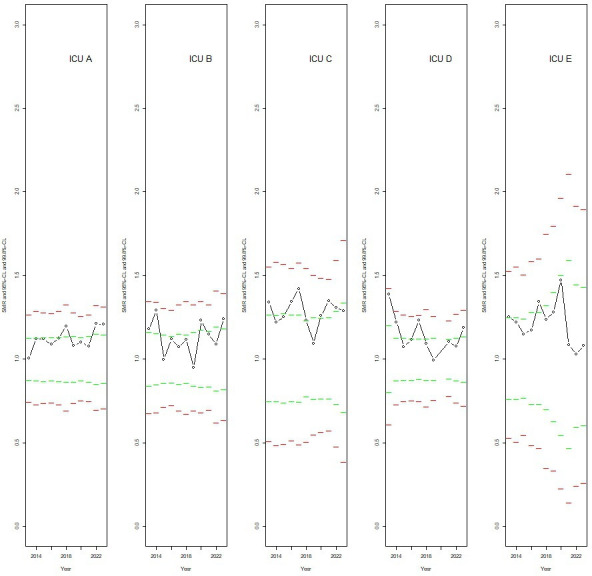
The SMRs of five ICUs in the highest quantile of the distribution of random intercepts as estimated in the logistic regression analysis restricted to the data of those ICU-calendar year combinations associated with an SMR within the 95% CL (variant ‘1. Narrow CL’), by calendar year. Shown are also the pertinent 95% CL (green dashed lines) and 99.8% CL (red dashed lines, including adjustment for overdispersion). CL, control limits; ICU, intensive care unit; SMR, standardised mortality ratio.

**Figure 2:**
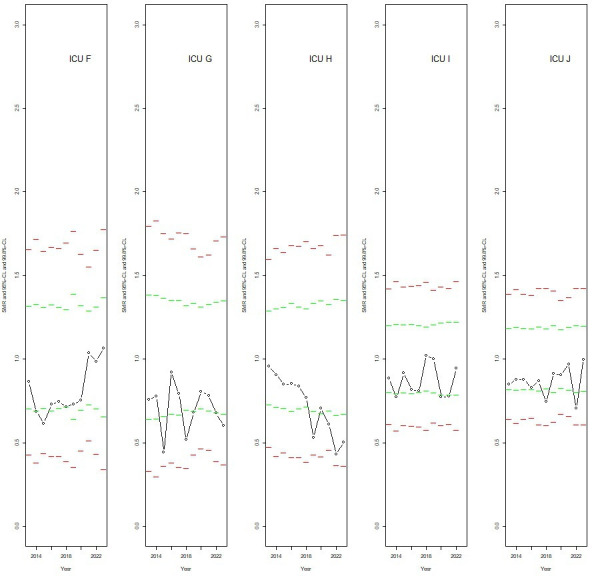
The SMRs of five ICUs in the lowest quantile of the distribution of random intercepts as estimated in the logistic regression analysis restricted to the data of those ICU-calendar year combinations associated with an SMR within the 95% CL (variant ‘1. Narrow CL’), by calendar year. Shown are also the pertinent 95% CL (green dashed lines) and 99.8% CL (red dashed lines, including adjustment for overdispersion). CL, control limits; ICU, intensive care unit; SMR, standardised mortality ratio.

In figures1  2, the available SMRs with the applicable 95% and 99.8% CL for a selection of ICUs are shown by calendar year. These ICUs contributed to the logistic regression analysis in variant ‘1. Narrow CL’. Figure 1 is related to five ICUs with a predicted random intercept in the highest 5% quantile of the distribution of random intercepts, and figure 2 to five ICUs with a predicted random intercept in the lowest 5% quantile. ICU-calendar year combinations with an SMR outside the 95% CL were not included in the logistic regression analysis for variant ‘1. Narrow CL’. However, these SMRs, if present, are shown here to make the picture for a particular ICU complete. The graphs show that the SMRs, even those within the narrowest 95% CL, are often consistently at the higher part (figure 1) or at the lower part (figure 2) of the funnel. In these graphs, all SMRs were within the widest 99.8% CL and, thus, these ICUs were not labelled as potential outliers for any calendar year when adopting the more flexible criteria.

## Discussion

This study showed an elevated MOR, which signals non-random systematic differences between ICUs, even when restricting the analysis to those ICU-calendar year combinations that were not labelled as potential outlier and when applying the narrowest 95% CL for the definition of outlier. Results of the logistic regression analysis for variant ‘1. Narrow CL’ (table 2 and online supplemental table 3) together with the graphical display (figure 1) showed that ICUs may have consistently increased SMRs for a number of subsequent years, but may stay within the 95% CL for one or more of these years. Thus, focusing on funnel plots for separate calendar years may miss this and give rise to the spurious impression that ICUs with an SMR within the 95% CL (or 99.8% CL) do perform well and are similar to other ICUs within these CL. Benchmarking should therefore address persistent variation, not just annual outlier detection. Our multi-level analysis with the MOR as an indicator of underlying performance heterogeneity may be helpful to identify ICUs with divergent mortality rates not explained by case mix.

### Interpretation

Funnel plots are based on traditional frequentist test theory, requiring sufficient evidence to allow rejection of the null hypothesis assuming a true ‘underlying’ SMR of 1.00. However, not rejecting a null hypothesis does not necessarily imply acceptance of that null hypothesis, as ‘absence of evidence’ is not identical with ‘evidence of absence’.^17^

By adjustment of the CL for overdispersion, the interpretation of a funnel plot as a series of well-defined formal tests against a null hypothesis of a global or locally defined benchmark is no longer fully sound.^18^ By allowing extra variance of the underlying performance, the CL also identifies those ICUs at the periphery of the underlying distribution of random effects. When interpreted in this way, we identified overdispersion twice. At first, when estimating a Winsorised estimate of the overdispersion factor for further widening of the 99.8% CL, this was done with the aim of showing what is done in the daily registry practice. The common interpretation is that variation among datapoints located within the CL (even when widened by adjustment for overdispersion) is due to chance and does not require further inspection. Second, we identified overdispersion by estimating the MOR, demonstrating that the standard interpretation needs nuance and that systematic non-random differences between ICUs may be present among ICUs labelled as ‘in control’. Expanding the CL by using the 99.8% probability limits, adjustment for possible overdispersion and applying an interpolation method that allows for p values lower than the nominal probability surely will limit the risk of false positive labelling of a hospital as a potential outlier. On the other hand, important variation between ICUs may be overlooked in the standard funnel plot interpretation, as even the analysis restricted to ICU-calendar year combinations with an SMR within the narrowest 95% CL showed significant differences between ICUs.

Multilevel methods that account for hospital-specific effects beyond a simplified dichotomy are needed to get a better impression of the performance of a hospital.^18^,^21^ Furthermore, breaking down such an analysis by different diagnostic subgroups, as was done in online supplemental table 4, will be helpful to find out for which subgroups an ICU performs better and for which it performs less.

### Strengths and limitations

In the present study, a large and high-quality database was used. The techniques of random-effects logistic regression, calculation of the MOR and graphical display can be easily implemented in the standard data processing for benchmarking.

For an individual ICU, the positions of the estimated SMR for the present year and for the 5 or 10 foregoing years, together with the CL relevant for each separate year, can be shown, as we did in figures1  2 for a selection of ICUs. Furthermore, we may report the ICU-specific random intercepts with their position in the distribution (ie, in terms of percentiles), both from a logistic model including all relevant calendar years and from models for separate calendar years.

This may be helpful for an ICU to evaluate their position in comparison with other ICUs and the persistence of this position in the course of recent years. In this way, the simplified categorisation of ‘in control’ versus ‘out of control’ can be transcended in practice. The issue of the validity of the categorisation in ‘potential outliers’ versus those that are ‘in control’ and not outliers when using different CL is a general statistical issue that may also raise issues for other datasets, registries and outcomes.

All analyses were adjusted for the APACHE-IV mortality risk, which was recalibrated on the data of each separate calendar year. The APACHE-IV model is a well-tried and tested method for individual prognosis of patients admitted to the ICU.^9^ However, the APACHE-IV model is still limited as domain-specific knowledge (such as neuro-anatomic location and extent in case of stroke) or life-restraining comorbidities such as chronic obstructive pulmonary disease are not included. This implies that case-mix correction is not perfect.

Therefore, a high SMR, even over several successive years, does not necessarily mean impaired quality of care, but requires further careful and detailed inspection. Stratifying the analysis by different diagnostic subgroups, as we did in online supplemental table 4, may be an important step in this examination. Furthermore, although data are subjected to quality checks and on-site audits, inaccuracies in data collection and registration cannot be ruled out. Possible systematic registration errors should be taken into account when considering outlying SMRs in detail.

Simulation studies are needed to find the distribution of the ‘real’ underlying performance among those ICUs who are labelled as ‘in control’ using CL with varying locations, and, thus, get better insight into the reliability of the associated classifications. Still, our results clearly show that using data across multiple calendar years will increase the signal-to-noise ratio and will provide a more stable estimate of the performance. Our study may be regarded as an important complement to those studies that focus primarily on the reliability of flagging organisations as high or low performers, by focusing on the other organisations labelled as ‘in control’.

In the present study, we established between-ICU variance in hospital mortality but did not investigate factors at the ICU level (such as volume, bed-occupancy or academic setting) that might explain differences between ICUs. ICUs with a persistent low mortality may be teaching examples for those ICUs with high mortality rates that strive for improvement. Furthermore, the MOR may be different for different subgroups of patients by age, gender or severity of illness and comorbidity. Taking these differences into account necessitates extension of the logistic regression model with a random slope and use of a more complicated formula for the MOR.^15^ These are topics for further research.

## Conclusions

We showed that information concerning the position of the SMR in the funnel plot for a separate calendar year is not sufficient to evaluate the quality of care of an ICU. Rather than relying solely on single-year classification, SMR interpretation should incorporate longitudinal trends to provide context and to assess room for improvement.

## Supplementary material

10.1136/bmjopen-2025-107572 online supplemental file 1

## Data Availability

Data are available upon reasonable request.

## References

[1] Hansen J, Ahern S, Earnest A (2023). Evaluations of statistical methods for outlier detection when benchmarking in clinical registries: a systematic review. BMJ Open.

[2] Power GS, Harrison DA (2014). Why try to predict ICU outcomes?. Curr Opin Crit Care.

[3] Seaton SE, Manktelow BN (2012). The probability of being identified as an outlier with commonly used funnel plot control limits for the standardised mortality ratio. BMC Med Res Methodol.

[4] Spiegelhalter DJ (2005). Funnel plots for comparing institutional performance. Stat Med.

[5] Massie AB, Segev DL (2013). Rates of false flagging due to statistical artifact in CMS evaluations of transplant programs: results of a stochastic simulation. Am J Transplant.

[6] Seaton SE, Barker L, Lingsma HF (2013). What is the probability of detecting poorly performing hospitals using funnel plots?. BMJ Qual Saf.

[7] National Intensive Care Evaluation (NICE) Foundation Data in beeld 2023.

[8] Verburg IW, Holman R, Peek N (2018). Guidelines on constructing funnel plots for quality indicators: A case study on mortality in intensive care unit patients. Stat Methods Med Res.

[9] Zimmerman JE, Kramer AA, McNair DS (2006). Acute Physiology and Chronic Health Evaluation (APACHE) IV: hospital mortality assessment for today’s critically ill patients. Crit Care Med.

[10] van de Klundert N, Holman R, Dongelmans DA (2015). Data Resource Profile: the Dutch National Intensive Care Evaluation (NICE) Registry of Admissions to Adult Intensive Care Units. Int J Epidemiol.

[11] Manktelow BN, Seaton SE (2012). Specifying the probability characteristics of funnel plot control limits: an investigation of three approaches. PLoS One.

[12] Spiegelhalter DJ (2005). Handling over-dispersion of performance indicators. Qual Saf Health Care.

[13] Austin PC, Merlo J (2017). Intermediate and advanced topics in multilevel logistic regression analysis. Stat Med.

[14] Merlo J, Chaix B, Ohlsson H (2006). A brief conceptual tutorial of multilevel analysis in social epidemiology: using measures of clustering in multilevel logistic regression to investigate contextual phenomena. J Epidemiol Community Health.

[15] Yarnell C, Pinto R, Fowler R (2019). Measuring variability between clusters by subgroup: An extension of the median odds ratio. Stat Med.

[16] Bates DM (2010). Lme4: mixed-effects modeling with R.

[17] van der Willik EM, van Zwet EW, Hoekstra T (2021). Funnel plots of patient-reported outcomes to evaluate health-care quality: Basic principles, pitfalls and considerations. Nephrology (Carlton).

[18] Abel GA, Agniel D, Elliott MN (2024). The good, the bad and the ugly: What do we really do when we identify the best and the worst organisations?. BMJ Qual Saf.

[19] Abel G, Elliott MN (2019). Identifying and quantifying variation between healthcare organisations and geographical regions: using mixed-effects models. BMJ Qual Saf.

[20] Kasza J, Moran JL, Solomon PJ (2013). Evaluating the performance of Australian and New Zealand intensive care units in 2009 and 2010. Stat Med.

[21] Solomon PJ, Kasza J, Moran JL (2000). Identifying unusual performance in Australian and New Zealand intensive care units from 2000 to 2010. BMC Med Res Methodol.

