# Supplementary material for: Mortality differences between ICUs that are regarded as ‘in control’: a longitudinal register-based study in the Netherlands, 2013–2023
Source: BMJ Open. 2026 Apr 20;16(4):e107572. doi: 10.1136/bmjopen-2025-107572 (PMC13110599; doi:10.1136/bmjopen-2025-107572)
Supplement: online supplemental file 1 [file bmjopen-16-4-s001.docx]

Supplement Figure 1. Selection of admissions for inclusion in the study


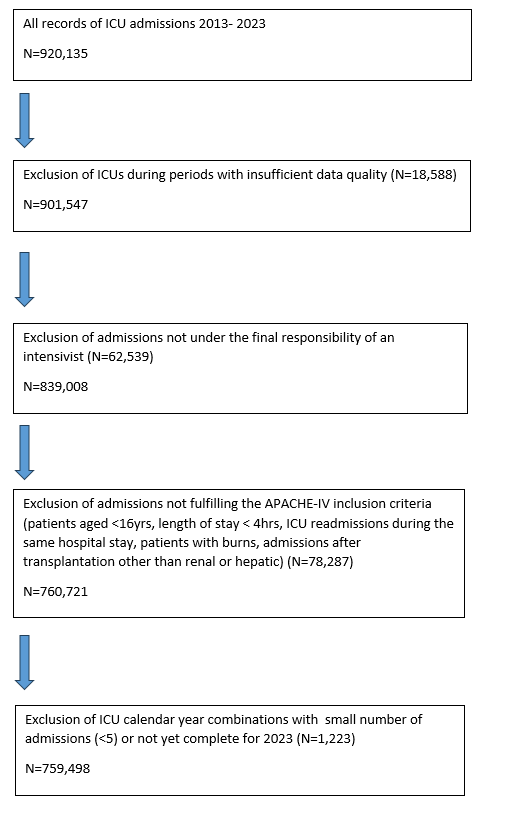


Supplement Figure 2. The SMRs related to all admission diagnoses in 2019 and the 95%- and 99.8%-Control Limits (CL).


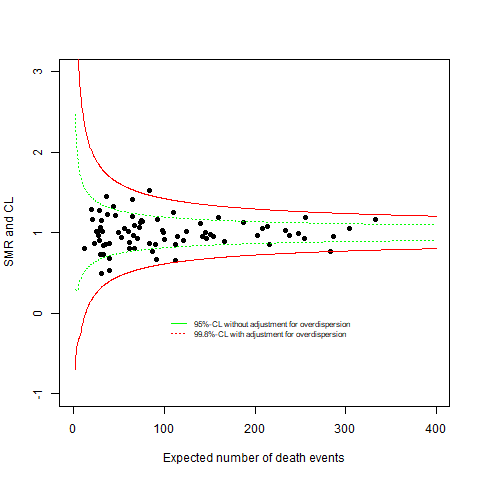


Supplement Table 1 Definition of diagnostic subgroups, based on one of the two registered APACHE-IV diagnoses that was regarded as the most important as reason for ICU admission

| Diagnostic subgroup |  |  |
| --- | --- | --- |
|  |  |  |
| Cardiac surgery group |  | C1 – Post-Operative – Cardiovascular 235 – 248, 268, 269, 275, 280, 226, 227, 231, 249, 252, 271, 272, 276  C1 – Post-Operative – Cardiovascular 232, 233, 258, 270, 251, 256 if referring specialism is cardiology (3), cardio-pulmonal surgery (29), or thorax surgery (50).  If one of the two registered APACHE IV diagnoses belongs to the cardio group, the patient’s admission is categorized as ‘Cardio’ if referring specialism is cardiology (3), cardio-pulmonal surgery (29), or thorax surgery (50). |
| Non-cardiac surgery group |  | All other ICU admission diagnoses |
|  |  |  |
| Specific diagnostic subgroups |  |  |
| Out-of-hospital Cardiac arrest  (OHCA) |  | M1 - Non-Operative – Cardiovascular, codes: 6 and/ or registered cardio-pulmonal resuscitation (cpr) and admission source is Emergency department of present (3) or other hospital (10) or home (15) |
| Community acquired pneumonia (CAP) |  | M1 – Non-operative – Cardiovascular, code: 38  M8 – Non-operative-respiratory, codes: 168, 170, 172, 446, 447, 448, 449, 450  AND time between hospital admission and ICU admission < 2 days  AND admission type is medical (1) and admission source is not from other hospital (8-14) |
| Sepsis |  | M1 – Non-Operative – Cardiovascular, codes: 34 – 40 |
| Trauma |  | M10 – Non-operative – Trauma, codes: 190 – 225  C10 – Post-operative – Trauma, codes: 410-445 |
| Stroke |  | M7 – Non-operative – Neurologic, codes: 125, 132, 148, 149 |
|  |  | C7 – Post-operative – Neurologic, code: 367 |
| Malignancy |  | M4 – Non-operative – Hematology, codes: 83 - 91 |
|  |  | M9 – Non-operative – Transplant, code: 178 |
|  |  | C9 – Post-Operative – Transplant, code: 398 |
|  |  | C4 – Post-Operative – Hematology, code 342-343 |
|  |  | M2 - Non-operative – Gastrointestinal, code: 52-56 |
|  |  | M3 – Non-operative – Genitourinary, code: 77 |
|  |  | M5 – Non-operative – Metabolic/ Endocrine, codes: 96, 108 |
|  |  | M7 – Non-operative – Neurologic, code: 136 |
|  |  | M8 – Non-operative – Respiratory, codes: 155-158 |
|  |  | C2 - Post-operative – Gastrointestinal, codes: 286-290,314, |
|  |  | C3 - Post-operative – Genitourinary, codes: 319, 324, 326,327, 330, 331, 338, 340 |
|  |  | C7 – Post-operative – Neurologic, codes: 369, 370, 375 |
|  |  | C8 - Post-operative – Respiratory, codes: 383, 384, 390, 391, 393 |
| Infections (no Sepsis, no CAP) |  | M1 - Non-Operative – Cardiovascular, code 18 |
|  |  | M2 - Non-operative – Gastro intestinal, codes: 57, 60, 63, 68 |
|  |  | M3 - Non-operative – Genitourinary, code: 76 |
|  |  | M6 - Non-operative – Musculoskeletal/ skin, codes: 112,114 |
|  |  | M7 - Non-operative – Neurologic, codes: 122, 127, 134, |
|  |  | M8 - Non-operative – Respiratory, codes: 167, 169, 171 |
|  |  | C2 - Post-operative Gastro-intestinal, codes: 292-294, 296, 298, 300, 310 |
|  |  | C6 - Post-operative Musculoskeletal/ skin, code: 346 |
|  |  | C7 - Post-operative – Neurologic, code: 354 |
|  |  | C8 - Post-operative – Respiratory, code: 386 |
| Cardio-vascular (no OHCA) |  | M1 - Non-operative – Cardiovascular, codes: 1-5, 7-17, 19-33, 41-46 |
|  |  |  |
| Gastro-intestinal (no malignancy, no infections) |  | M2 - Non-operative – Gastrointestinal, codes: 47 – 51, 58, 59, 61, 62, 64-67, 69, 303 |
| Metabolic/ endocrine |  | M5 – Non operative – Metabolic/ Endocrine, codes: 94, 95, 97-105 |
| Neurology (no stroke, no malignancy, no infections) |  | M7 - Non-Operative- Neurologic, codes: 123, 124, 126, 128-131, 133, 135, 137-147 |
| Respiratory (no CAP, no malignancy, no infections) |  | M8 – non-operative – Respiratory, codes: 150-154, 159-166, 173-177 |
| Other diagnoses, medical |  | All other diagnoses when admission type is medical (1) |
|  |  |  |
| Other diagnoses, emergency surgery |  | All other diagnoses when admission type is emergency surgery (2) |
| Other diagnoses, elective surgery |  | All other diagnoses when admission type is elective surgery (4) |
|  |  |  |

Supplement Table 2. See Table 1. The numbers and percentages related to all diagnoses were broken down by calendar year in 2013 - 2023

|  |  |  | Categories for SMR delineated by the CL | | | | |
| --- | --- | --- | --- | --- | --- | --- | --- |
|  | Total |  | <99.8%-CL  low  N (%) | <95%-CL low  N (%) | >95%-CL low - <95%-CL high  N (%) | >95%-CL high  N (%) | > 99.8%-CL high  N (%) |
| All diagnoses |  |  |  |  |  |  |  |
|  |  |  |  |  |  |  |  |
| 2013 ICUs N (row %) | 82 |  | 1 (1.2) | 9 (11) | 61 (74.4) | 11 (13.4) | 0 (0) |
| 2013 Admissions N (row %) | 73,219 |  | 1,410 (1.9) | 12,226 (16.7) | 49,395 (67.5) | 10,188 (13.9) | 0 (0) |
| 2013 Hospital death N (col %) | 8,819 |  | 80 (5.7) | 1,261 (10.3) | 6,077 (12.3) | 1,401 (13.8) | 0 (0) |
| 2014 ICUs N (row %) | 83 |  | 2 (2.4) | 11 (13.3) | 61 (73.5) | 9 (10.8) | 0 (0) |
| 2014 Admissions N (row %) | 77,423 |  | 3,864 (5) | 11,787 (15.2) | 46,420 (60) | 15,352 (19.8) | 0 (0) |
| 2014 Hospital death N (col %) | 8,839 |  | 298 (7.7) | 1,124 (9.5) | 5,385 (11.6) | 2,032 (13.2) | 0 (0) |
| 2015 ICUs N (row %) | 85 |  | 2 (2.4) | 8 (9.4) | 65 (76.5) | 10 (11.8) | 0 (0) |
| 2015 Admissions N (row %) | 76,951 |  | 4,281 (5.6) | 8,150 (10.6) | 50,180 (65.2) | 14,340 (18.6) | 0 (0) |
| 2015 Hospital death N (col %) | 9,118 |  | 249 (5.8) | 744 (9.1) | 6,310 (12.6) | 1,815 (12.7) | 0 (0) |
| 2016 ICUs N (row %) | 85 |  | 1 (1.2) | 9 (10.6) | 65 (76.5) | 9 (10.6) | 1 (1.2) |
| 2016 Admissions N (row %) | 76,474 |  | 1,390 (1.8) | 11,385 (14.9) | 50,826 (66.5) | 11,081 (14.5) | 1,792 (2.3) |
| 2016 Hospital death N (col %) | 9,047 |  | 79 (5.7) | 974 (8.6) | 6,369 (12.5) | 1,380 (12.5) | 245 (13.7) |
| 2017 ICUs N (row %) | 84 |  | 1 (1.2) | 11 (13.1) | 62 (73.8) | 10 (11.9) | 0 (0) |
| 2017 Admissions N (row %) | 73,598 |  | 2,272 (3.1) | 11,154 (15.2) | 51,125 (69.5) | 9,047 (12.3) | 0 (0) |
| 2017 Hospital death N (col %) | 8,763 |  | 168 (7.4) | 1,057 (9.5) | 6,186 (12.1) | 1,352 (14.9) | 0 (0) |
| 2018 ICUs N (row %) | 78 |  | 0 (0) | 12 (15.4) | 58 (74.4) | 6 (7.7) | 2 (2.6) |
| 2018 Admissions N (row %) | 68,316 |  | 0 (0) | 14,523 (21.3) | 44,772 (65.5) | 6,133 (9) | 2,888 (4.2) |
| 2018 Hospital death N (col %) | 8,546 |  | 0 (0) | 1,272 (8.8) | 5,984 (13.4) | 851 (13.9) | 439 (15.2) |
| 2019 ICUs N (row %) | 77 |  | 1 (1.3) | 7 (9.1) | 61 (79.2) | 7 (9.1) | 1 (1.3) |
| 2019 Admissions N (row %) | 67,413 |  | 2,378 (3.5) | 6,780 (10.1) | 49,812 (73.9) | 7,673 (11.4) | 770 (1.1) |
| 2019 Hospital death N (col %) | 7,984 |  | 216 (9.1) | 450 (6.6) | 5,970 (12) | 1220 (15.9) | 128 (16.6) |
| 2020 ICUs N (row %) | 76 |  | 0 (0) | 8 (10.5) | 62 (81.6) | 6 (7.9) | 0 (0) |
| 2020 Admissions N (row %) | 62,399 |  | 0 (0) | 7,038 (11.3) | 49,868 (79.9) | 5,493 (8.8) | 0 (0) |
| 2020 Hospital death N (col %) | 8,264 |  | 0 (0) | 771 (11) | 6,557 (13.1) | 936 (17) | 0 (0) |
| 2021 ICUs N (row %) | 75 |  | 0 (0) | 8 (10.7) | 60 (80) | 7 (9.3) | 0 (0) |
| 2021 Admissions N (row %) | 59,386 |  | 0 (0) | 6,348 (10.7) | 48,266 (81.3) | 4,772 (8) | 0 (0) |
| 2021 Hospital death N (col %) | 8,240 |  | 0 (0) | 657 (10.3) | 6,754 (14) | 829 (17.4) | 0 (0) |
| 2022 ICUs N (row %) | 77 |  | 0 (0) | 10 (13) | 59 (76.6) | 8 (10.4) | 0 (0) |
| 2022 Admissions N (row %) | 61,871 |  | 0 (0) | 9,144 (14.8) | 43,393 (70.1) | 9,334 (15.1) | 0 (0) |
| 2022 Hospital death N (col %) | 7,773 |  | 0 (0) | 773 (8.5) | 5,621 (13) | 1,379 (14.8) | 0 (0) |
| 2023 ICUs N (row %) | 75 |  | 0 (0) | 7 (9.3) | 57 (76) | 11 (14.7) | 0 (0) |
| 2023 Admissions N (row %) | 62,448 |  | 0 (0) | 7,996 (12.8) | 40,618 (65) | 13,834 (22.2) | 0 (0) |
| 2023 Hospital death N (col %) | 7,435 |  | 0 (0) | 598 (7.5) | 5,072 (12.5) | 1,765 (12.8) | 0 (0) |
|  |  |  |  |  |  |  |  |

Supplement Table 3. See Table 3. The logistic regression analysis including all admission diagnoses is performed for each separate calendar year 2013 - 2024.

|  | variant ‘1. Narrow CL’ | | variant ‘2. Expanded CL’ | | variant ‘3. All’ | |
| --- | --- | --- | --- | --- | --- | --- |
|  |  |  |  |  |  |  |
|  | MOR | [95%-CI] | MOR | [95%-CI] | MOR | [95%-CI] |
| All admission diagnoses |  |  |  |  |  |  |
|  |  |  |  |  |  |  |
| 2013 | 1.13 | [1.08-1.191] | 1.204 | [1.156-1.265] | 1.221 | [1.172-1.284] |
| 2014 | 1.14 | [1.09-1.202] | 1.23 | [1.179-1.295] | 1.253 | [1.201-1.321] |
| 2015 | 1.12 | [1.073-1.177] | 1.236 | [1.182-1.307] | 1.253 | [1.198-1.324] |
| 2016 | 1.137 | [1.091-1.194] | 1.232 | [1.181-1.3] | 1.263 | [1.208-1.334] |
| 2017 | 1.122 | [1.075-1.18] | 1.242 | [1.188-1.313] | 1.248 | [1.194-1.319] |
| 2018 | 1.136 | [1.088-1.196] | 1.252 | [1.198-1.324] | 1.292 | [1.232-1.37] |
| 2019 | 1.122 | [1.073-1.181] | 1.224 | [1.169-1.295] | 1.243 | [1.188-1.316] |
| 2020 | 1.119 | [1.074-1.176] | 1.221 | [1.167-1.292] | 1.221 | [1.167-1.292] |
| 2021 | 1.132 | [1.089-1.188] | 1.213 | [1.162-1.281] | 1.213 | [1.162-1.281] |
| 2022 | 1.098 | [1.054-1.153] | 1.237 | [1.182-1.31] | 1.237 | [1.182-1.31] |
| 2023 | 1.135 | [1.079-1.206] | 1.267 | [1.204-1.351] | 1.267 | [1.204-1.351] |
|  |  |  |  |  |  |  |

Supplement Table 4. See Table 3. The analysis including all calendar years 2013 – 2023 is performed for separate diagnostic subgroups.

|  | variant ‘1. Narrow CL’ | | variant ‘2. Expanded CL’ | | variant ‘3. All’ | |
| --- | --- | --- | --- | --- | --- | --- |
|  |  |  |  |  |  |  |
|  | MOR | [95%-CI] | MOR | [95%-CI] | MOR | [95%-CI] |
| 2013-2024 |  |  |  |  |  |  |
|  |  |  |  |  |  |  |
| Out-of-hospital cardiac arrest (OHCA) | 1.201 | [1.137-1.282] | 1.349 | [1.269-1.458] | 1.349 | [1.269-1.458] |
| Community-acquired Pneumonia (CAP) | 1.111 | [1.073-1.155] | 1.229 | [1.182-1.289] | 1.232 | [1.185-1.292] |
| Sepsis | 1.171 | [1.129-1.225] | 1.284 | [1.229-1.355] | 1.295 | [1.24-1.369] |
| Trauma | 1.074 | [1-1.157] | 1.212 | [1.124-1.335] | 1.212 | [1.124-1.335] |
| Stroke | 1.036 | [1-1.114] | 1.189 | [1.123-1.275] | 1.196 | [1.131-1.282] |
| Malignancy | 1.312 | [1.243-1.404] | 1.43 | [1.343-1.549] | 1.43 | [1.343-1.549] |
| Infections (no sepsis, no CAP) | 1.132 | [1.092-1.181] | 1.24 | [1.19-1.304] | 1.25 | [1.199-1.315] |
| Cardiovascular (no OHCA) | 1.088 | [1.047-1.132] | 1.171 | [1.126-1.23] | 1.172 | [1.126-1.23] |
| Gastro-intestinal disorders | 1.091 | [1-1.161] | 1.271 | [1.203-1.359] | 1.278 | [1.21-1.367] |
| Metabolic disorders | 1 | [1-1.192] | 1.131 | [1-1.376] | 1.131 | [1-1.376] |
| Neurologic disorders (no stroke) | 1.098 | [1-1.192] | 1.309 | [1.219-1.433] | 1.309 | [1.219-1.433] |
| Respiratory disorders (no CAP) | 1.09 | [1.043-1.137] | 1.22 | [1.172-1.282] | 1.224 | [1.175-1.287] |
| Other diagnoses | 1.094 | [1-1.221] | 1.33 | [1.22-1.479] | 1.33 | [1.22-1.479] |
| Surgery emergency | 1.072 | [1-1.138] | 1.258 | [1.193-1.345] | 1.26 | [1.195-1.347] |
| Surgery elective | 1.205 | [1.141-1.288] | 1.384 | [1.291-1.515] | 1.396 | [1.301-1.528] |
|  |  |  |  |  |  |  |
