# Supplementary material for: Mortality differences between ICUs that are regarded as ‘in control’: a longitudinal register-based study in the Netherlands, 2013–2023
Source: BMJ Open. 2026 Apr 20;16(4):e107572. doi: 10.1136/bmjopen-2025-107572 (PMC13110599; doi:10.1136/bmjopen-2025-107572)
Supplement: Supplementary file 2 [file bmjopen-2025-107572.reviewer_comments.pdf]

PEER REVIEW HISTORY

BMJ Open publishes all reviews undertaken for accepted manuscripts. Reviewers are asked to complete a checklist review form and are provided with free text boxes to elaborate on their assessment. These free text comments are reproduced below.

This paper was submitted to another journal from BMJ but declined for publication following peer review. The authors addressed the reviewers' comments and submitted the revised paper to BMJ Open. The paper was subsequently accepted for publication at BMJ Open.

ARTICLE DETAILS

Title (Provisional)

Mortality differences between ICUs that are regarded as 'in control': a longitudinal register-based study in the Netherlands, 2013-2023

Authors

Termorshuizen, Fabian; Brinkman, Sylvia; Arbous, Mendi S.; Dongelmans, Dave; de Keizer, Nicolette; Bakhshi-Raiez, Ferishta

---

VERSION 1 - REVIEW

**Reviewer**	**1**
**Name**	**Reviewer 1**
**Affiliation**	
**Date**	**28-May-2025**
**COI**	

Comments not published pending reviewer's consent

VERSION 2 - REVIEW

**Reviewer**	**2**
**Name**	**Kelson, Zoe**
**Affiliation**	**University of Exeter, Mathematics**
**Date**	**12-Nov-2025**
**COI**	**None**

This study examined whether statistically significant and relevant differences in mortality over time between ICUs regarded as ‘in control’ are present.

Reviewer comments:

"Data on ICU admissions (2013-2023) registered in the National Intensive Care Evaluation (NICE) registry were used." [Abstract]

and

"Since 2016, all Dutch ICUs participate...

All admissions recorded in the NICE database with admission dates in the years 2013-2023 were extracted. The data of ICUs related to periods of insufficient data quality, as determined by onsite data quality audits, were excluded. Furthermore, those admissions not under the final responsibility of an intensive care physician (such as recovery or CCU patients) and admissions not fulfilling the APACHE-IV inclusion criteria were excluded (see Suppl. Figure 1) "

Can the authors please further comment on the completeness of the included data?

"Demographic, clinical and laboratory data are collected to make calculation of the Acute Physiological And Chronic Health Evaluation (APACHE) IV mortality risk and case-mix adjustment possible (9). ICU- and hospital length of stay and ICU- and hospital death are included in the registry. "

Can the authors please confirm if there was any missing data?

"877 ICU-calendar year combinations were included, covering 759,498 unique admissions. "

Can the authors please comment on whether the included cohort can be considered to be representative?

"Two pairs of CL were calculated. The first pair was based on the 95% probability limits of a Poisson distribution with the expected number of deaths (x-axis) as the parameter for the definition of both the expectation and the variance (6). As the Poisson distribution is related to discrete numbers, the exact positions of the upper and lower 95%-CL were determined by applying an interpolation method. For the 95%-CL, the interpolation resulting in a narrower funnel was used by allowing P values higher than the nominal probability of 5% (for details, see (11)). The second pair of CL was based on the 99.8% probability limits (6). For these limits, the interpolation method resulting in a wider funnel was used by allowing P values less than the nominal probability of 0.2% (for details, see (11)). Possible overdispersion, i.e. when the variance appears to be higher than expected given a Poisson distribution, was taken into account by calculating a so-called Winsorised estimate of the variance inflation factor (4, 12). This was done for each separate calendar year and, if higher than 1.00, used to further expand the 99.8%-CL for that calendar year by multiplying the variance with this factor...

In the analysis of the total ICU population the overdispersion factor appeared to be higher than 1.00 in all calendar years and the 99.8%-CL were adjusted (widened) accordingly. However, for specific diagnostic subgroups this factor was often lower than 1.00, indicating underdispersion (i.e., the variance was lower than expected under the assumption of a Poisson distribution). If so, the 95%-CL, not the 99.8%-CL, was adjusted (narrowed) accordingly for that specific diagnostic subgroup "

The analytical approach has been clearly described by the authors.

"This was first performed for ICU-calendar year combinations with an SMR within the narrowest 95%-CL. Next, the selection was expanded including ICUs with an SMR within the 99.8%-CL adjusted for overdispersion. Finally, all ICUs were included."

A number of insightful analyses have been explored.

"Hospital death was analyzed in a logistic regression model including the APACHEIV mortality risk, calendar year, and a random intercept for ICU. The random intercept variance was translated to the Median Odds Ratio (MOR). " [Abstract]

and

"Hospital mortality as outcome was analyzed in a logistic regression model on individual-level patient data with adjustment for the APACHE-IV mortality risk (categorized in quintiles) and calendar year, and ICU included as random intercept...

This method implies a refitting of the model multiple times with different values for the estimated parameters, resulting in a range of values that are more or are less likely as judged by the Likelihood ratio test."

Appropriate modelling methods have been applied.

Confidence intervals are suitably communicated in the Results.

"Adjustment with the APACHE-IV model is still limited as disorder-specific knowledge is not included. Therefore, a high SMR, even over several successive years, does not necessarily mean impaired quality of care, but requires further careful and detailed inspection such as stratifying the analysis by different diagnostic subgroups, as we did in Suppl. Table 4. In addition, possible systematic registration errors should be taken into account when considering outlying SMRs in detail. Simulation studies are needed to find the distribution of the 'real' underlying performance among those ICUs who are labelled as 'in control' using CL with varying locations, and, thus, get better insight into the reliability of the associated classifications. "

Can the discussion on the study limitations please be expanded on?

Thanks for providing a copy of the STROBE checklist.

Reviewer 3

Name **Secombe, Paul J**

Affiliation **Alice Springs Hospital, ICU**

Date **06-Jan-2026**

COI **Member of ANZICS CORE Management Committee (which also oversees ICU benchmarking)**

Mortality differences between ICUs that are regarded as ‘in control’: a challenge to standard funnel plot interpretation

Thank you for the opportunity to review this interesting manuscript from the NICE Registry in the Netherlands. Benchmarking ICU performance is a core function of registries worldwide, and this paper highlights a fundamental limitation of using funnel plots to identify “potential” outlying units based on SMR. It is a valuable addition to the literature in this area.

The study draws on high-quality registry data from NICE, which is internationally respected, covering 88 ICUs over an 11-year period.

Global Comments

I am not a biostatistician, and a statistical review would be helpful. Furthermore, the manuscript is very data-heavy and wording could be refined to improve accessibility for clinicians.

Despite this, it is an important piece of work that adds meaningfully to benchmarking literature.

The language is occasionally challenging. For example, funnel plots do not “find” ICUs but rather identify those with a statistical signal suggesting higher-than-expected risk-adjusted mortality—relative either to peers (if CIs reflect SMR variance) or to an SMR of 1 (if CIs reflect predicted deaths). This signal should prompt further evaluation using approaches such as Mohamad’s pyramid. To me, these ICUs should be considered potential outliers , as variation may reflect random chance, data quality, case mix, resources, processes of care, or clinician practice.

Consider using terms such as “potential high outlier” (above the 99% CI) and “potential low outlier” (below the CI), rather than “in control” or “out of control,” which carry negative connotations. The term “potential” acknowledges that outlier status may result from chance or correctable data issues, and detailed analysis is required before drawing conclusions.

Specific Comments

1. Abstract

Abstract conclusion : Consider rewording as: “Assessment of ICU mortality performance should integrate both cross-sectional funnel plots, longitudinal SMR trends and MOR to better capture persistent patterns of excess risk.”

Implications for research/practice: Suggest: “Rather than relying solely on single-year classification, SMR interpretation should incorporate longitudinal trends to provide context and complement cross-sectional analyses.”

2. Introduction:

Emphasise that hospital mortality (not ICU mortality) is benchmarked.

Clarify SMR definition to highlight risk adjustment: I suggest “The standardised mortality ratio (SMR), defined as the ratio of observed deaths to expected deaths—where expected deaths are derived from risk-adjusted models accounting for illness severity and case mix for a given ICU in a specific calendar year—is commonly used as the primary benchmarking metric.”

Correct typographical errors (second paragraph of introduction, paragraph in methods describing MOR).

Replace pejorative terms like “underperforming” - for example “The potential reputational impact on clinicians and hospitals publicly identified as potential high mortality outliers is significant.”

3. Methods

Why was calendar year chosen rather than another 12-month block (e.g., financial year)? Presumably because NICE publishes annual funnel plots—please clarify.

4. Results:

For a data-heavy manuscript, the results section feels thin. If word count allows, highlight key findings (e.g., MOR values and their interpretation).

The tables are not well integrated into the text..

5. Discussion:

The first sentence is confusing. To me, the paper demonstrates that an elevated MOR signals non-random, systematic differences between ICUs, even when SMRs fall within control limits. Benchmarking should therefore address persistent variation, not just annual outlier detection. Funnel plots may miss this. MOR should complement funnel plots as an indicator of underlying performance heterogeneity, helping identify ICUs with persistently higher mortality unexplained by case mix or illness severity. Could the authors comment?

Replace “0” with “null” in the second paragraph.

How would/could MOR and/or longitudinal SMR trends be incorporated into routine benchmarking activity?

VERSION 2 - AUTHOR RESPONSE

Reviewer: 2
Prof. Zoe Kelson, University of Exeter

Comments to the Author:
This study examined whether statistically significant and relevant differences in mortality over time between ICUs regarded as 'in control' are present.

Response
Thank you for thoughtfully reviewing our manuscript and positive comments. Please, find our answers to the points you raised below.

Reviewer comments:

"Data on ICU admissions (2013-2023) registered in the National Intensive Care Evaluation (NICE) registry were used." [Abstract]
and
"Since 2016, all Dutch ICUs participate...
All admissions recorded in the NICE database with admission dates in the years 2013-2023 were extracted. The data of ICUs related to periods of insufficient data quality, as determined by onsite data quality audits, were excluded. Furthermore, those admissions not under the final responsibility of an intensive care physician (such as recovery or CCU patients) and admissions not fulfilling the APACHE-IV inclusion criteria were excluded (see Suppl. Figure 1) "

Can the authors please further comment on the completeness of the included data?

"Demographic, clinical and laboratory data are collected to make calculation of the Acute Physiological And Chronic Health Evaluation (APACHE) IV mortality risk and case-mix adjustment possible (9). ICU- and hospital length of stay and ICU- and hospital death are included in the registry. "

Can the authors please confirm if there was any missing data?

Response
Since 1996, the start of the NICE registry, the number of ICUs participating in the NICE registry grew steadily and from 2016 onwards, all admissions to all Dutch ICUs are recorded in the NICE registry. For this study, we included all recorded admissions in the years 2013-2023, so a few ICU-calendar year combinations were missing. In Table 2, the number of included ICUs increased from 82 in 2013 to 85 in 2016. However, these number should be interpreted with caution for evaluation of completeness, as some ICUs were discontinued, went through a merger process and/ or different ICUs at different locations but belonging to the same hospital were put together. As written in the Methods section: To ensure that the

data are of high quality, the data are subjected to automated quality checks, onsite data quality audits take place, and data collectors participate in training sessions (#Ref 10). Therefore, the recorded admissions were complete for date of admission, hospital of admission, hospital survival status (that is, dead or alive at the time of hospital discharge), and for the presence of an estimate for the APACHE-IV probability score. There are some missing data on individual items of the APACHE-IV model, but this is taken into account using the guideline for calculation of the APACHE-IV. For example, laboratory data may be missing because there was no reason to examine this, e.g. for Bilirubin when there is no clinical indication for failure of liver function. In that case, zero points may be assigned. Likewise, if data on a comorbid condition is lacking, zero points were assumed.

Modification of manuscript
We added to the Methods section (paragraph Data) the following cursive sentences:
In 1996, the NICE registry started the collection of data on ICU admissions in the Netherlands. *When an ICU participates, all its admissions are recorded.*
And: *In case of missing data on individual items of the APACHE-IV model, this was taken into account using the guideline for calculation of the APACHE-IV. Often missingness was due to lack of clinical indication to assess a lab test, and zero points should be assigned for that item.*

___
"877 ICU-calendar year combinations were included, covering 759,498 unique admissions. "
Can the authors please comment on whether the included cohort can be considered to be representative?

Response
We feel that the included cohort can be considered fully representative for the ICU benchmark practice in the Netherlands as the selection steps performed (see Suppl Figure 1) are in accordance with the selection usually done when assessing ICU performance for individual calendar years. As ICUs and critically care patients in the Netherlands are the scope of the study, we cannot assume without question that our results are applicable to ICU patients in other countries and with other health care systems. Still, our main message of challenging the standard funnel plot interpretation has probably broad significance. Therefore, the Discussion / Strengths and Limitations section include the sentence: The issue of the validity of the categorization in ‘potential outliers’ versus those that are ‘in control’ and not outliers when using different CL is a general statistical issue that also may raise for other datasets, registries and outcomes.

___
"Two pairs of CL were calculated. (…) If so, the 95%-CL, not the 99.8%-CL, was adjusted (narrowed) accordingly for that specific diagnostic subgroup "
The analytical approach has been clearly described by the authors.

Response
Thank you for your favorable feedback

___
"This was first performed for ICU-calendar year combinations with an SMR within the narrowest 95%-CL. Next, the selection was expanded including ICUs with an SMR within the 99.8%-CL adjusted for overdispersion. Finally, all ICUs were included."
A number of insightful analyses have been explored.

Response
Thank you for your favorable feedback.

___
"Hospital death was analyzed in a logistic regression model including the APACHEIV mortality risk, calendar year, and a random intercept for ICU. The random intercept variance was translated to the Median Odds Ratio (MOR). " [Abstract]
and
"Hospital mortality as outcome was analyzed in a logistic regression model (…) by the Likelihood ratio test."
Appropriate modelling methods have been applied.

Confidence intervals are suitably communicated in the Results.

Response
Thank you for your favorable feedback.
___
"Adjustment with the APACHE-IV model is still limited as disorder-specific knowledge is not included. Therefore, a high SMR, even over several successive years, does not necessarily mean impaired quality of care, but requires further careful and detailed inspection such as stratifying the analysis by different diagnostic subgroups, as we did in Suppl. Table 4. In addition, possible systematic registration errors should be taken into account when considering outlying SMRs in detail. Simulation studies are needed to find the distribution of the 'real' underlying performance among those ICUs who are labelled as 'in control' using CL with varying locations, and, thus, get better insight into the reliability of the associated classifications. "

Can the discussion on the study limitations please be expanded on?

Response
We thank the reviewer for this useful suggestion. The paragraph in the Discussion section on study limitations was extended with a number of topics.

Modification of manuscript
In the second paragraph of the section Discussion/ Strengths and Limitations:
- we elaborated on the use of the APACHE-IV model for – case-mix correction,
- on inaccuracies and errors in data collection,
- on further research concerning factors associated with the variance between hospitals and estimation of the MOR for different subgroups.

Reviewer: 3
Dr. Paul J Secombe, Alice Springs Hospital
Comments to the Author:
Mortality differences between ICUs that are regarded as ‘in control’: a challenge to standard funnel plot interpretation

Thank you for the opportunity to review this interesting manuscript from the NICE Registry in the Netherlands. Benchmarking ICU performance is a core function of registries worldwide, and this paper highlights a fundamental limitation of using funnel plots to identify “potential” outlying units based on SMR. It is a valuable addition to the literature in this area.

The study draws on high-quality registry data from NICE, which is internationally respected, covering 88 ICUs over an 11-year period.

Response
Thank you for thoughtfully reviewing our manuscript and the recommendation. Please, find our answers to your global and specific comments below.

___
Global Comments

I am not a biostatistician, and a statistical review would be helpful.

Response
After the first submission of our manuscript to BMJ Quality & Safety we obtained an elaborate review of a statistician. All comments of this review were taken into account in the new version of the manuscript that was submitted to BMJ Open. This review and our response (including the modifications of the earlier version of the manuscript) were included in the submission to BMJ Open. We hope that this will be sufficient for you.

__
Furthermore, the manuscript is very data-heavy and wording could be refined to improve accessibility for clinicians.

Response & Modification of the manuscript
We modified the text of the manuscript to make it more accessible for clinicians. We followed your suggestions below.

E.g., in the Introduction and Methods section, the statistical terms ‘in-‘ versus ‘out-of-control’ were explained in terms of ‘potential outlier’, and it was emphasized that further examination is needed to find the reasons before clinically interpreting an ICU as ‘underperformer’.
In the introduction, it was explained how the expected numbers of deaths were derived from a risk-adjustment model.
The text of the Results section was extended with better explanation of the data from the Tables.
The first paragraph and Conclusions of the Discussion section was rephrased according to your suggestions.

We expanded on a number of topics in the Discussion section. See also our answer to the comments of Reviewer #1.

___
Despite this, it is an important piece of work that adds meaningfully to benchmarking literature.

Response
Thank you for your favorable feedback.

___
The language is occasionally challenging. For example, funnel plots do not “find” ICUs but rather identify those with a statistical signal suggesting higher-than-expected risk-adjusted mortality—relative either to peers (if CIs reflect SMR variance) or to an SMR of 1 (if CIs reflect predicted deaths). This signal should prompt further evaluation using approaches such as Mohamad’s pyramid. To me, these ICUs should be considered potential outliers , as variation may reflect random chance, data quality, case mix, resources, processes of care, or clinician practice.

Consider using terms such as “potential high outlier” (above the 99% CI) and “potential low outlier” (below the CI), rather than “in control” or “out of control,” which carry negative connotations. The term “potential” acknowledges that outlier status may result from chance or correctable data issues, and detailed analysis is required before drawing conclusions.

Response
Thank you for the useful suggestion. Indeed, ‘out of control’ does not necessarily imply divergent quality of care, whereas ‘in control’ does not mean that there is no room for improvement (which is actually the main topic of our study). In the new version, we addressed this issue and the interpretation of ‘in-’ vs ‘out of control’ as ‘potential high outlier’ vs ‘potential low outlier’. However, the terms ‘in-‘ vs ‘out of control’ and ‘outlier’ are general terminology in the field of statistical science on benchmarking and quality monitoring (eg, see Spiegelhalter, Statist Med 2005; 24: 1185-1202), and interpreted as neutral descriptive-statistical, not pejorative, terms. Therefore, we feel that we cannot fully remove these terms from the manuscript, but have explained them where needed and changed them where possible.

Modification of manuscript
In Abstract, Introduction, Methods, Results, and Discussion, the terms of ‘in control’ vs. ‘out of control’ are explained and completed with the term ‘potential high/ low outlier’.

___
Specific Comments
1. Abstract
Abstract conclusion : Consider rewording as: “Assessment of ICU mortality performance should integrate both cross-sectional funnel plots, longitudinal SMR trends and MOR to

better capture persistent patterns of excess risk.”

Implications for research/practice: Suggest: “Rather than relying solely on single-year classification, SMR interpretation should incorporate longitudinal trends to provide context and complement cross-sectional analyses.”

Response
Thanks for the good suggestions. The first one was incorporated in the conclusion of the Abstract. The second one was incorporated in the Conclusion at the end of Discussion section.

___
2. Introduction:
Emphasise that hospital mortality (not ICU mortality) is benchmarked.

Clarify SMR definition to highlight risk adjustment: I suggest “The standardised mortality ratio (SMR), defined as the ratio of observed deaths to expected deaths— where expected deaths are derived from risk-adjusted models accounting for illness severity and case mix for a given ICU in a specific calendar year—is commonly used as the primary benchmarking metric.”

Correct typographical errors (second paragraph of introduction, paragraph in methods describing MOR).

Replace pejorative terms like “underperforming” - for example “The potential reputational impact on clinicians and hospitals publicly identified as potential high mortality outliers is significant.”

Response
Thank you for the good suggestions. They were all incorporated in the new version of the manuscript. ‘… Publicly labelled as underperformers …’ was replaced by ‘… as outliers …’. In the sentence thereafter ‘…underperformer…’ was replaced by ‘… potential underperformer…’ to make better clear the reason for reputational damage.

___
3. Methods
Why was calendar year chosen rather than another 12-month block (e.g., financial year)? Presumably because NICE publishes annual funnel plots—please clarify.

Response
Yes, indeed, NICE publishes always in calendar time blocks, mainly as calendar years, and the aim of the study was to follow the usual registry practice of NICE and many other quality registries.

Modification in manuscript

This was added as clarification to the Methods section, subsection Statistical analysis, paragraph The Standardized Mortality Ratio (SMR), after the first sentence: *Calendar year was chosen as the unit of reporting time, as the aim of the study was to follow the usual registry practice of NICE (and many other quality registries).*

___
4. Results:
For a data-heavy manuscript, the results section feels thin. If word count allows, highlight key findings (e.g., MOR values and their interpretation).

The tables are not well integrated into the text..

Response
We elaborated the Results section with more details and references to the Tables in the main manuscript and in the Supplement.

___
5. Discussion:
The first sentence is confusing. To me, the paper demonstrates that an elevated MOR signals non-random, systematic differences between ICUs, even when SMRs fall within control limits. Benchmarking should therefore address persistent variation, not just annual outlier detection. Funnel plots may miss this. MOR should complement funnel plots as an indicator of underlying performance heterogeneity, helping identify ICUs with persistently higher mortality unexplained by case mix or illness severity. Could the authors comment?

Response
We feel that you give a very good summary of the results and main message of our study. Indeed, the MOR signals non-random systematic differences between ICUs and benchmarking may address this persistent variation. This was strengthened in the new version of the manuscript.

Modification of manuscript
The first paragraph of the Discussion section was partly rephrased according to your suggested summary: This study showed an elevated MOR, which signals non-random systematic differences between ICUs, even when restricting the analysis to those ICU-calendar year combinations that were not labelled as potential outlier and when applying the narrowest 95%-CL for the definition of outlier.

___
Replace “0” with “null” in the second paragraph.

Response
Thank you, we followed your suggestion in the new version of the manuscript.

___
How would/could MOR and/or longitudinal SMR trends be incorporated into routine benchmarking activity?

Response
Thank you for this important point for the Discussion section. At first, we may report to an individual ICU the SMR for the year in question with the SMRs of that ICU for the 5 or 10 previous calendar years, together with the matching Control Limits for each specific calendar year. An example of this strategy is presented in Figure 1.A and Figure 1.B in which 2023 may be regarded as the index year of the report and the years 2013-2022 as the preceding comparison years. In these figures, this was restricted to those ICUs (N=10) with an estimate of their random intercept at the extremes (5% and 95%-percentiles) of the distribution, but we may do this for all ICUs. Furthermore, we may report the ICU specific random intercepts with their position in the distribution (that is, in terms of percentiles), both from a logistic regression model including all relevant calendar years and from separate models for separate calendar years. When explanatory notes are added to such report, this may be helpful for an ICU to evaluate their position in comparison with other ICUs and the persistence of this position in the course of recent calendar years, without dichotomizing this position as ‘in-‘ versus ‘out of control’. The MOR in itself as metric, indicative of between-hospital variation in general, is probably not useful for monitoring of individual ICUs.

Modification of manuscript
We elaborated on this issue in the first paragraph of the section Discussion, paragraph Strengths and Limitations:
For an individual ICU, the positions of the estimated SMR for the present year and for the 5 or 10 foregoing years together with the CL relevant for each separate year can be shown, as we did in Figure 1A and 1.B for a selection of ICUs. Furthermore, we may report the ICU specific random intercepts with their position in the distribution (that is, in terms of percentiles), both from a logistic model including all relevant calendar years and from models for separate calendar years. This may be helpful for an ICU to evaluate their position in comparison with other ICUs and the persistence of this position in the course of recent years. In this way, the simplified categorization of ‘in control‘ versus ‘out of control’ can be transcended in practice.

VERSION 3 - REVIEW

**Reviewer**	**3**
**Name**	**Secombe, Paul J**
**Affiliation**	**Alice Springs Hospital, ICU**
**Date**	**18-Mar-2026**
**COI**	

The authors have provided responses to all areas of concern
